# Informing the development and uptake of a weight management intervention for preconception: a mixed-methods investigation of patient and provider perceptions

**DOI:** 10.1186/s40608-017-0144-6

**Published:** 2017-02-06

**Authors:** Samantha M. Harden, NithyaPriya S. Ramalingam, Kathryn E. Wilson, Emily Evans-Hoeker

**Affiliations:** 1 Department of Human Nutrition, Virginia Tech Foods, and Exercise, 1981 Kraft Dr, Blacksburg, VA USA; 2 Department of OBGYN, Virginia Tech Carilion School of Medicine, 1231 S. Jefferson St, Roanoke, VA 24013 USA; 3 Virginia Tech Translational Biology, Medicine, and Health Program, 1981 Kraft Dr, Blacksburg, VA USA

**Keywords:** Translation, Weight-loss, Clinical, Preconception

## Abstract

**Background:**

It is recommended for women to have a healthy body mass index before conception. However, there is limited research on appropriate preconception interventions for weight loss. Furthermore, there is a lack of knowledge on providers’ willingness to refer to particular behavioral interventions and the degree to which patients would attend those interventions.

**Methods:**

A cross-section of 67 patients and 21 providers completed surveys related to their demographics and willingness to refer/attend a number of interventions for weight loss. A case study of three patients from the target audience was used to elicit detailed feedback on preconception weight status and weight loss intervention.

**Results:**

Overall, patients were willing to attend a variety of interventions, regardless of BMI category. Focus group participants shared that weight loss prior to conception would be beneficial for them and their child, but cited barriers such as time, location, and the way providers encourage weight loss. Providers were willing to refer to a number of behavioral interventions, and were less willing to prescribe weight loss medications than other intervention options.

**Conclusions:**

A number of intervention strategies may be well received by both patients and providers in preconception care to assist with weight loss prior to conception. Future research is needed on intervention effects and sustainability.

**Electronic supplementary material:**

The online version of this article (doi:10.1186/s40608-017-0144-6) contains supplementary material, which is available to authorized users.

## Background

Implementing behavioral weight management interventions in preconception care may improve maternal and fetal health outcomes [1]. Preconception care, as part of a broader health care model, aims to provide health promotion, screening, and interventions for all women of reproductive age to reduce risk factors that might affect future pregnancies [2]. However, there is little evidence on the effects of preconception health promotion or its existence in practice, despite recommendations for routinized population-level preconception health promotion [3, 4]. One difficulty encountered in clinical practice is that women do not routinely present to a health care practitioner prior to conception for the purposes of preparing for conception. Rather, women often wait until after conception to present for obstetrical care.

Given that many reproductive aged women do not have healthcare practitioners other than their obstetrician or gynecologist (OBGYN), and the expectation that healthy lifestyle interventions may be translated into preconception care [5, 6], it is arguable that such interventions should be a focus of routine OBGYN care. Integrating theory-based behavior change programs offered in community settings through clinical referral efforts can promote successful weight control in healthy, non-pregnant patient samples [7, 8]. However, the lack of consensus on the appropriate type, frequency, and delivery of preconception care complicates these efforts [9–11]. A series of systematic reviews documented little evidence to support the effectiveness of brief counseling for healthful eating, physical activity, and weight control in a clinical setting [12–14]. More work is needed to determine the optimal dose, type, and delivery personnel to maximize preconception weight management care (including both weight loss and healthy weight maintenance) within clinical settings.

As providers are key determinants in clinical intervention uptake, it is imperative to describe characteristics of interventions to which providers would most likely refer patients as well as the characteristics of providers themselves [15]. That is, the individual characteristics of providers (e.g., their own health status, years in their position) may influence their support of an intervention. It is equally necessary to describe intervention features that will be attractive to the target audience (i.e., overweight/obese women prior to conception) [16]. There is preliminary evidence that patients who plan to become pregnant are twice as likely to commit to behavior change strategies than those who are already pregnant [5]; however, this study did not describe the role of intervention preferences such as intervention frequency, location, mode, and delivery personnel (e.g., trainer versus physician). The purpose of this study was to describe the patient and provider perceptions of weight management interventions to provide preliminary support for the implementation of lifestyle interventions in preconception care.

## Methods

We used a cross-sectional, sequential, mixed-methods design [17] to collect data regarding the perceptions of weight management interventions among OBGYN patients and providers. Separate surveys were developed and administered to participating patients and providers. A focus group was conducted with patients. Quantitative data were used to describe characteristics of patients and providers as well as characteristics of interventions preferred by patients and those more likely to be recommended by providers. Qualitative data elucidated overweight/obese patients’ perceptions of weight management, while open-ended responses from providers related to their physical activity and dietary recommendations. Consent was implied with the returned anonymous surveys and written consent was obtained from patients prior to the start of the focus group. The Carilion Clinic Institutional Review Board approved this study.

### Setting

Nine clinics within a 60-mile radius of Roanoke, VA were eligible for participation in the study; eight agreed to recruit from their offices for the patient portion of the study. In total, these clinics see 400 new patients, with a range from 18 to 70 new encounters, per month. Patients attending these medical visits pay for services via Medicaid (32%), Anthem (28%), commercial insurance (20%), self-pay (13%), and other (7%), including Medicare). The clinic that declined to participate was located in Blacksburg, VA and has a similar payor mix and sees approximately 50 new encounter patients per month.

### Sample

#### Patients

Patients presenting to a general Carilion Clinic OBGYN office or the infertility clinic between January 5^th^ and March 31^st^, 2015, who were between 21 and 35 years of age, were eligible to participate. Patients did not need to be currently pregnant or intending to become pregnant to participate. No exclusions were made based upon patient BMI, to allow for a description of patient preferences for those in need of preconception weight loss interventions (i.e., overweight and obese patients), as well as those in need of preconception weight maintenance (i.e., normal weight patients). The invitation for patients to participate in the study was extended by front desk staff who were informed of the study and its eligibility criteria. The intake/registration for the visit was used to determine if the patients met the age inclusion criteria. Information about the opportunity to participate in a focus group related to preconception weight management healthcare was described at the end of the paper-distributed survey. Patients who indicated interest in conceiving within the next 12 months and had a body mass index (BMI) ≥ 25.0 were eligible to share contact information for participation in a focus group.

#### Providers

Carilion Clinic OBGYN faculty and mid-level providers were eligible to complete the care provider online survey between January 25^th^ and February 11^th^, 2015. Providers of urogynecology, gynecologic oncology, and maternal fetal medicine were excluded due to their lack of patients in the target population. In addition, the only provider from the infertility clinic was excluded due to conflict of interest.

### Measures

Demographic variables (age, race, ethnicity, marital status, employment status, education level, and socioeconomic status) were collected in accordance with Census data questions. BMI (kg/m^2^) was calculated from self-reported height and weight for both providers and patients. Please see Additional files 1 and 2 for complete patient and provider survey tools. 

#### Patients

One item was used to assess self-reported health status on a 4-point forced-answer scale of ‘Extremely Healthy’ to ‘Extremely Unhealthy’; including a ‘Don’t know’ option. Moderate-to-vigorous physical activity level (MVPA) was assessed using the Godin Leisure Time Exercise Questionnaire [18]. Patients were asked to identify the physical activity recommendations for Americans, and were asked to indicate their physical activity level in the context of national recommendations (‘Less than recommended amount’, ‘Meeting recommendations’, ‘More than the recommended amount’, ‘I do not engaged in physical activity’, ‘Unsure’). To assess self-efficacy for physical activity, patients were asked to rate their confidence level for engaging in moderate intensity physical activity for 30 min, 5 or more days per week’ using a 5-point Likert scale from ‘not at all’ to ‘completely’ confident.

Patients were asked about their likeliness to attend interventions based on (1) duration (‘30 min’, ‘60 min’, ‘90 min’, or ‘Would not attend’), (2) frequency (‘3 times per week’, ‘Weekly’, ‘Monthly’, or ‘Would not attend’) and (3) type (‘In-person’, ‘Online’, ‘Via email’, ‘Via DVD/Video’, ‘Via text message’, or ‘Would not attend’). One item queried whether participants ‘would need an incentive (e.g., gift card, door prize) to attend a health promotion class’ using a 5-point Likert scale from ‘strongly agree’ to ‘strongly disagree’. To determine intervention content that would be attractive to this population, a list of 21 evidence-based strategies for behavior change were presented (e.g., cooking demonstrations, opportunities to interact with a group, exercise diary). These response options were listed following the item stem: “The following program characteristics would be appealing to me in a health promotion program. (Please check all that apply).” The preconception weight management interventions proposed were based on behavioral interventions that have previously resulted in clinically meaningful weight loss [19–23] and/or improvements in physical activity [24].

Patients who were eligible for the focus group were invited to attend and elaborate on their perceptions of weight status and weight management interventions. The interview guide (Additional file 3) was based on the Theory of Planned Behavior [25] (i.e., subjective norms, perceived behavioral control, and attitude toward physical activity/healthy eating behaviors). The full semi-structured interview guide is available upon request. Due to the low sample size of focus group participants, the results of the qualitative data are presented as an exploratory case study.

#### Providers

Providers indicated their position (‘Attending physician,’ ‘Resident Physician,’ ‘Nurse Practitioner,’ or ‘Other’) and number of years at the targeted non-profit healthcare clinic (open ended response). Two items were used to assess provider perceptions of their 21–35 year old non-pregnant patients: (1) perceptions of the patients’ health status were rated using 4-point forced-answer scale of ‘Extremely healthy’ to ‘Extremely unhealthy’, including a ‘Don’t know’ option, and (2) confidence in the patients’ ability to engage in moderate intensity physical activities for 30 min, 5 or more days per week was rated on a 5-point Likert scale from ‘not at all’ to ‘completely’ confident. It was hypothesized that there would be congruence between providers’ perceptions of patients’ ability to meet physical activity recommendations and their perceptions of health.

Providers were asked to rate their willingness to refer to 14 interventions, such as individualized diet/activity plan or weight loss medications, using a 5-point Likert scale (‘Strongly Agree’ to ‘Strongly Disagree’). These interventions’ characteristics aligned with interventions evidenced to result in clinically meaningful weight loss or improvements in physical activity [19–24]. Lastly, providers were asked to indicate their current recommendations for diet and physical activity via an open-ended response item asking “In your non-pregnant 21–35 year old patients, what are your typical recommendations for [physical activity/diet]?”

### Analytical plan

#### Quantitative

Statistical analyses were conducted using SPSS v. 20.0 (IBM, 2012). Means and standard deviations of continuous variables and frequencies and proportions of nominal variables were calculated for the samples overall, as well as according to BMI group. In the patient sample, likelihood-ratio chi-square tests were used to identify significant differences between BMI groups in participant demographics, knowledge of physical activity recommendations, physical activity self-efficacy, physical activity level and preferences for a number of intervention features. Significant effects were further assessed using adjusted residuals, with a critical value of |1.96|. Variables for which participants could make more than one selection (e.g. mark all that apply), such as preferred class type, location, and exercise setting were coded dichotomously to indicate whether each participant had selected the respective feature or not, then analyzed independently for associations with BMI status. Univariate analysis of variance (ANOVA) was used to test for mean differences in age, number of times pregnant, number of live births, and total minutes of MVPA according to BMI status, as well as preferences in class frequency and duration, and self-reported need for incentives. A repeated measures ANOVA was used to compare mean scores of self-reported patient interest in a number of possible intervention methods/strategies. Pairwise comparisons were used to describe significant differences in the frequency of selection of intervention methods/strategies. To control for family-wise error, a Bonferroni adjusted *p*-value of .001 was used as a post hoc control for multiple comparisons in the patient sample.

Mean differences in provider age and years at Carilion Clinic according to BMI status were tested with univariate ANOVA. Limited cell counts precluded an analysis of provider outcomes according to provider characteristics. Spearman’s rank order correlation was used to test the relationship between provider perceptions of the health of their non-pregnant 21–35 year old patients, and their confidence in the ability of these patients to engage in MVPA. Ratings for each strategy were standardized to a z-distribution and compared to the mean rating to identify recommendations which providers are significantly more or less willing (*p* < .05) to provide for weight-loss.

#### Qualitative

Trained research assistants transcribed the patient focus group audio-recording verbatim in Microsoft Word. Patient data were analyzed as a case study by the first and second authors using a deductive approach that aligned with constructs of Theory of Planned Behavior [25]. An inductive approach was used to determine salient themes related to intervention characteristics. All data are reported as a narrative [26] in chronological order of the interview guide. The first and second author reviewed provider open-ended responses for common themes.

## Results

### Patients

During the 6-month recruitment period, 67 patients volunteered to complete the paper/pencil distributed survey. These women had a mean(sd) age of 27.81(4.26) years, and were predominantly Caucasian (77%), married (61%), and employed for wages (75%). Descriptive statistics of patient characteristics are reported for the total sample as well as for groups categorized by BMI status (Table 1). BMI groups did not significantly differ in age, race, ethnicity, education, income, employment status, MVPA, number of pregnancies and live births, or patient type (general or infertility).

**Table 1:** Summary of patient characteristics

	Total sample	Normal weight	Overweight	Obese
*N*(%)	67 (100)	20 (29.9)	17 (25.4)	30 (46.2)
	M(sd)			
Age	27.81 (4.26)	26.80 (4.07)	26.82 (4.59)	29.03 (4.04)
Times pregnant	1.80 (1.86)	1.32 (1.67)	2.41 (1.97)	1.77 (1.87)
Live births	.92 (1.21)	.42 (.61)	1.29 (1.40)	1.03 (1.30)
Minutes of moderate-vigorous physical activity	215.36 (618.38)	119.56 (152.33)	436.04 (1118.10)	171.03 (456.96)
	*N*(%)			
Race				
White	52 (77.6)	18 (90.0)	10 (58.8)	24 (80.0)
Black/AA	10 (14.9)	1 (5.0)	5 (29.4)	4 (13.3)
Asian	1 (1.5)	1 (5.0)	0	0
Other	2 (3.0)	0	1 (5.9)	1 (3.3)
White and AA	2 (3.0)	0	1 (5.9)	1 (3.3)
Ethnicity				
Hispanic	1 (1.5)	0	0	1 (3.3)
Not Hispanic	55 (82.1)	14 (70.0)	15 (88.2)	26 (86.7)
Not sure	4 (6.0)	3 (15.0)	0	1 (3.3)
Education				
Grades 9-11	3 (4.5)	0	1 (5.9)	2 (6.7)
High School	20 (29.9)	6 (30.0)	8 (47.1)	6 (20.0)
Some college	18 (26.9)	3 (15.0)	5 (29.4)	10 (33.3)
College grad	18 (26.9)	8 (40.0)	3 (17.6)	7 (23.3)
Post college	8 (11.9)	3 (15.0)	0	5 (16.7)
Employment Status				
Employed	50 (74.6)	17 (85.0)	11 (64.7)	22 (73.3)
Self-employed	1 (1.5)	0	0	1 (3.3)
Out of work >1 year	3 (4.5)	0	2 (11.8)	1 (3.3)
Out of work <1 year	1 (1.5)	1 (5.0)	0	0
Home maker	6 (9.0)	2 (10.0)	3 (17.6)	1 (3.3)
Student	2 (3.0)	0	1 (5.9)	1 (3.3)
Disabled	1 (1.5)	0	0	1 (3.3)
Employed and a student	1 (1.5)	0	0	1 (3.3)
Self-employed homemaker	2 (3.0)	0	0	2 (6.7)
Income				
< 15,000	20 (29.9)	7 (35.0)	9 (52.9)	4 (13.3)
15,000-29,999	17 (25.4)	1 (5.0)	5 (29.4)	11 (36.7)
30,000-49,999	9 (13.4)	4 (20.0)	1 (5.9)	4 (13.3)
50,000-90,999	16 (23.9)	4 (20.0)	2 (11.8)	10 (33.3)
1000,000 or more	4 (6.0)	3 (15.0)	0	1 (3.3)
Marriage Status				
Single	19 (28.4)	5 (25.0)	9 (52.9)	5 (16.7)
Married	41 (61.2)	13 (65.0)	6 (35.3)	22 (73.3)
Separated	1 (1.5)	0	0	1 (3.3)
Divorced	2 (3.0)	0	1 (5.9)	1 (3.3)
Living common law	4 (6.0)	2 (10.0)	1 (5.9)	1 (3.3)
Infertility patient	26 (38.8)	11 (55.0)	2 (11.8)	13 (43.3)

Cases with missing values were included in proportion calculation

Sixty-three percent of the patients self-reported that they were ‘somewhat healthy’ when compared to others their age. Perceptions of health status were not significantly related to BMI status. Participants’ perception of their own weight status was accurate among half (52.5%) of those that responded to that survey item (n = 61); 45.9% underestimated their weight status. Perceptions of weight status were related to BMI status (*χ* ^2^(df) = 27.326(4); *p* < .001) such that normal weight participants were most likely, and obese participants were least likely to be accurate. Further, obese participants were more likely than others to underestimate their current weight-status.

A little more than half (57%) of the sample was able to correctly identify physical activity recommendations for adults. Fifty-two percent of the patient sample was very-to-completely confident that they could engage in these recommendations (‘moderate physical activities (e.g., not exhausting, light perspiration) for 30 min for 5 or more days per week’); whereas one individual was not at all confident and 20% were ‘somewhat confident.’ A total of 42(62.7%) participants indicated that they get less than the recommended amount, with 6% indicating they did not engage in any physical activity. BMI was not significantly related to any of these variables.

**Fig. 1:**
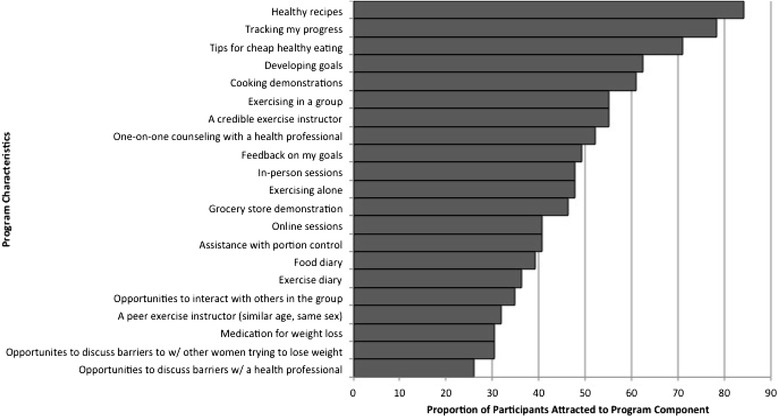
Proportion of participants attracted to proposed program content

Obese patients were more likely to want to attend a class at the gym than their overweight or normal weight counterparts, and overweight participants were significantly less likely to exercise at the gym when compared to their normal or obese counterparts (*χ* ^2^(df) = 13.963(2); *p* = .001). There was no significant relationship between BMI status and preferred class frequency or duration, nor was there for self-reported need for incentive. Proportions of the sample that indicated interest in specific strategies are rank-ordered and displayed in Fig. 1. Interest in specific strategies was not related to BMI status. When asked which of 21 strategies appealed to patients, the sample selected “healthy recipes” significantly more frequently than they did 18 of the 20 other strategies. “Tracking my progress” and “tips for cheap, healthy eating” were second and third most frequently selected methods, each being selected significantly more frequently than 17 and 13, respectively, of the other 20 strategies. Conversely, “opportunities to discuss barriers to success with a health professional was least frequently selected, and significantly so in comparison to half of the other strategies. Second and third least likely to be selected were “medication for weight-loss” and “opportunities to discuss barriers with other women trying to lose weight,” which were significantly less likely to be selected than six and nine of the other 20 strategies, respectively.

#### Patient case study

Eight of the 67 participants who completed the survey agreed to participate in a focus group (0.2%), and three attended. All three focus group participants were married and were categorized with an overweight/obese BMI. Participant 2 and 3 indicated their annual household income to be in the $50-90,000 range whereas Participant 1 reported $30-49,000. Two of the women were White (Participant 1 and 2) and one was Black (Participant 3). One participant had become pregnant (Participant 1) between the time of the survey and the focus group interview. All three participants had been diagnosed with previous conditions: hypertension (Participant 1 and 2), pre-diabetes (Participant 2), and Polycystic Ovary Syndrome (PCOS) (Participant 3). The focus group centered around three constructs of the Theory of Planned Behavior: subjective norms (i.e., an individuals perception about a particular behavior, which is influenced by the judgment of significant people in their life), perceived behavioral control (i.e., an individuals perceived ease or difficulty of performing a particular behavior) and attitude (i.e., an individual’s positive or negative evaluation of self-performance of the particular behavior).

#### Subjective norms

All three participants shared that the credibility of a healthcare professional-- whether a personal trainer, healthcare employee, or family member with expertise—influences their perception of support and encouragement. For example, Participant 2 stated*, “I would probably like (health education) best out of my doctor’s office cause that’s…who I trust. So I feel like they would be giving me the right information.”* However, Participant 1 and 2 spoke about the barriers to healthy changes based on their perceptions of provider’s recommendations:

*Participant 1:* “*[The physician was] just very rude and I understood what they were trying to say, but it was the way it came across.”*

*Participant 2 “I had a similar experience with a nurse practitioner. And I think one of the difficult things about when you’re not getting pregnant, it’s gotta be more than [my weight]. Because people bigger than me getting pregnant as well.”*

#### Perceived behavioral control

Although the participants noted benefits of losing weight, participants made comments related to the veracity of the BMI categories (based on a BMI chart provided for them to view): *“Participant 2: I feel like it’s unrealistic for me. To lose that amount of weight.”* While Participant 3 shared concerns: *“As far as saying that I’m obese, for me, I don’t feel that way. But I guess according to the [BMI I am]… I wouldn’t look right, weighing 140 pounds. It wouldn’t be possible for me, I don’t think.”*

When prompted about what would need to happen to convince them to lose weight prior to pregnancy, Participant 2 shared that: *“The convincing is not the problem. I know that I should, I’m convinced that I should…The blood pressure medication convinced me. The pre-diabetic convinced me.”*

The participants expressed a lack of time as a barrier to healthy lifestyle choices and behaviors and role modeling as reasons to be healthier.

*Participant 2: “I’ve got to make some time for myself because I give to everybody all day and so…I’m actually looking forward to telling people to leave me alone cause I have something (exercise) to do for me.”*

*Participant 3: “My thing is scheduling too. I have a 45 min commute one way to work.”*

#### Attitude toward PA/healthy eating behaviors

When prompted to discuss what might happen if they lost weight prior to pregnancy, Participant 3, who suffers from PCOS was not sure if weight loss would improve the likelihood that she would conceive: *“I don’t know necessarily my case if it would actually help me even get pregnant. Cause I have PCOS, so they say. So I don’t know that – I mean but I know losing weight and managing that can help the symptoms of that.”* The other two participants cited positive benefits of reducing the impact of current conditions, for example Participant 2 stated: *“It would definitely help me because I have high blood pressure, I’m pre-diabetic, so I need to lose the weight before…I get pregnant. So I know that it would make me healthier, and if I’m healthier then the baby would be healthier.”* All three participants noted that there are no negative effects of eating healthfully; e.g., “*Participant 3: I can’t think of any. No negatives.”* However, they did concur that weight must be lost safely, via their conversation:

*“Participant 3: You mean like if you rapidly lose it, you rapidly gain it back. At least I do.*

*Participant 1: Well, yeah, well it depends on how you lose it. It depends on --*

*Participant 2: If you starve yourself.”*

#### Intervention components

When prompted about the structure and content of potential healthy lifestyle intervention programs, the participants suggested the program meet after five in the evening and lasts for 6 months:

Participant 2: *“It’s enough time to develop a habit. And if you’re seeing the results. So, let’s say you go 3 months, but then month four, there’s no change then month five, there’s no change. Then there’s a conversation you can have while you’re still in the group like now what else do I need to do? Cause a lot of people hit a plateau -- can hit a plateau and you -- so what now? So I mean, it could be modified cause I think six months would be enough time to modify something*.”

The participants also spoke about the benefit of accountability that may be resultant group sessions:

*Participant 3: And that there’s somebody who cares that I -- if I’m doing it or if I’m not there. Like, would they say, “Oh, she didn’t even show up”.*

*Participant 2: “Part of being with a group is just that everybody’s got a common goal; everybody’s trying to do the same thing and you’re not by yourself, and that’s important for me.”*

Finally, as for the personal characteristics of the individual delivering the program, participants shared that credible health professionals need to be encouraging. Participant 3 preferred a female instructor while two of the participants preferred someone who has been overweight at some point in their life (Participant 1 and 3).

### Providers

Sixty-eight percent (*n* = 21) of eligible providers participated in the survey. Providers had a mean(sd) age of 43(10.1) years, and were mostly Caucasian (78%) females (60%). A majority of the respondents had worked at Carilion Clinic for 10.3(±9.9) years and 60% were attending physicians. Forty percent of the respondents were within the normal-weight BMI, though 30% did not provide height/weight data necessary to calculate BMI. Provider BMI groups did not differ in age or years at Carilion Clinic (*p* > .05).

Seventy-five percent of the providers indicated that their non-pregnant patients were “somewhat healthy” and that they were “moderately confident” that their patients can meet the national physical activity recommendations. There was no significant correlation between providers’ perceptions of patients’ health status and ability to meet the aerobic physical activity recommendations (rho = -.046; *p* = .855). A summary of health care provider characteristics is displayed in Table 2.

**Table 2:** Descriptive statistics for the total provider sample, and according to provider weight class

	Total sample *N* = 23	Missing BMI data *N*(%) = 7(30.4)	Normal weight *N*(%) = 9(39.1)	Overweight *N*(%) = 3(13.0)	Obese *N*(%) = 4(17.4)
	M (sd)				
Age	43.57 (10.11)	44.80 (16.78)	41.33 (6.40)	45.00 (11.36)	46.00 (9.13)
Years at Carilion	10.25 (9.87)	9.2 (7.69)	7.54 (9.81)	16.67 (16.07)	11.50 (8.88)
BMI (kg/m^2^)	26.58 (5.76)	N/A	22.45 (1.64)	27.71 (1.51)	35.02 (3.17)
	*N*(%)				
Provider Type					
Resident Physician	1 (4.3)	1 (14.3)	0	0	0
Nurse-Midwife	1 (4.3)	0	0	0	1 (25.0)
Nurse Practitioner	5 (21.7)	0	2 (22.2)	0	3 (75.0)
Attending Physician	14 (60.9)	4 (57.1)	7 (77.8)	3 (100)	0
Gender					
Female	14 (60.9)	3 (42.9)	5 (55.6)	2 (66.7)	4 (100)
Male	7 (30.4)	2 (28.6)	4 (44.4)	1 (33.3)	0
Race					
White	18 (78.3)	4 (57.1)	9 (100)	1 (33.3)	4 (100)
Asian	2 (8.7)	0	0	2 (66.7)	0
Not sure	2 (4.3)	1 (14.3)	0	0	0
Ethnicity					
Hispanic	2 (8.7)	4 (57.1)	1 (11.1)	1 (33.3)	4 (100)
Not Hispanic	18 (78.3)	0	8 (88.9)	2 (66.7)	0
Not sure	1 (4.3)	1(14.3)	0	0	0
Patient health ratings					
*Non-pregnant 21-35 yo*					
Extremely healthy	2 (8.7)	1 (14.3)	1 (11.1)	0	0
Somewhat healthy	15 (65.2)	3 (42.9)	6(66.7)	3 (100)	3 (75.0)
Not healthy	3 (13.0)	0	2 (22.2)	0	1 (25.0)
*Low risk pregnant pts*					
Extremely healthy	3 (13.0)	1 (14.3)	2 (22.2)	0	0
Somewhat healthy	11 (47.8)	1 (14.3)	5 (55.6)	2 (66.7)	3 (75.0)
Not healthy	2 (8.7)	0	1 (11.1)	0	1 (25.0)

*yo* years old, *pts* patients

**Fig. 2:**
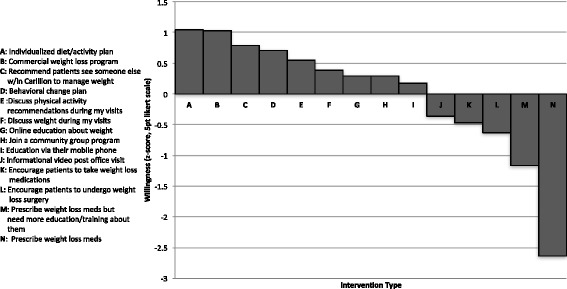
Ratings of provider willingness to discuss, prescribe, recommend or encourage intervention, ranked and standardized to a z-distribution

Providers were willing to recommend a variety of behavioral interventions: informational videos, community group programs, online education, a mobile-application educational program, a behavior change plan, referral to someone within Carilion to further discuss weight management, and commercial programs (e.g., Weight Watchers). Providers were most willing to recommend patients receive an individualized diet/activity plan, though this difference was not statistically significant (*p* = .150). Providers were significantly less willing to recommend weight-loss medications than the other options (*p*=,029). See Fig. 2 for standardized-and-ranked recommendation ratings.

#### Open-ended responses

Sixteen providers shared open-ended responses related to physical activity recommendations and 10 provided a response related to diet. Overall, the providers (*n* = 16) recommended moderate activity 30-40 min, four to five times a week, in line with current physical activity recommendations. Provider (*n* = 10) messaging for nutrition and diet was mixed, with some providers recommending: “*increased fruit and vegetables*” or “*low carbohydrates*” and others including lists of recommendations such as “*fruits, vegetables, lean meats, low- or no-fat dairy, whole grains, lean protein*.”

## Discussion

This mixed-methods study was developed to identify patient and provider perceptions of weight management interventions that could be widely implemented in an OBGYN practice with the goal of optimizing weight loss or maintaining healthy weight in patients engaged in preconception healthcare. Data were collected to describe patients’ characteristics and willingness to attend a variety of behavioral interventions, as well as providers’ characteristics and their willingness to recommend a variety of behavioral interventions. This descriptive report can inform intervention development and implementation by elucidating intervention characteristics that may fit within care practices, and appeal to patient and provider preferences in order to improve clinical outcomes. A number of behavior change techniques have been used for weight loss interventions [27] and providing preliminary support for program fit may speed the translation of the intervention into sustained practice [28].

Only 57% of the patients in our study were able to correctly identify the formal recommendation for physical activity and 62.7% indicated they were not meeting these recommendations. This could be due to the fact that almost half (45.9%) of the patients in our study underestimated their weight status, and possibly did not recognize the need for physical activity, or that only 52% of the patients felt confident that they could adhere to recommendations. These findings suggest that a first step in improving preconception weight management may involve educating patients on their weight status, as well as current physical activity recommendations.

Most patients, including those who were normal weight, reported that they would attend an intervention with sessions that met monthly (42%) or weekly (25%). Conversely, 27% reported that they would not attend for any frequency, and two (3%) patients (both of whom were obese) indicated that they would attend classes that met three times per week. There were no significant differences by BMI on class location, contact type, or duration. These results reject the hypothesis that different intervention components may be more effective for different demographics (i.e., for what subset of individuals does an intervention [not] work) [29], but rather that these intervention types may be well-received by all individuals.

However, the focus group with obese individuals provided additional information related to their perceptions. Obese individuals who want to become pregnant may benefit from face-to-face and group-based interventions to collectively overcome barriers and provide social support. [30] Focus group participants shared positive attitudes about group-based sessions encouraging attendance and progress. This affinity for in-person sessions is contrary to previous literature which indicates that participants in obesity treatment programs often perceive attending in-person treatment sessions as burdensome [31]. This disconnect from obese patients’ preferences for weight management in a prior systematic review [31] and the preferences of the participants in this study indicates more work is needed to determine the transferability and generalizability of obese patients’ preferences specific to preconception care.

The focus group responses, based on the theory of planned behavior, supported an increased focus on addressing attitudes toward the effects of weight management and the perceived judgments from providers. Patients reported mixed attitudes towards weight management and its effects on conception, providing an opportunity for patient education on the relationship between weight optimization and conception. Although from a limited sample, the barriers (e.g., time, lack of knowledge) reported in this case study provide potential targets to increase intentions and self-efficacy for weight management. Patients preferred providers who shared similar characteristics (i.e., female and personal experiences with managing weight) indicating that provider characteristics play a role in weight management interventions. With these patient preferences in mind, more formative work is needed to determine the likelihood of intervention adoption among providers across settings [32].

Providers representing nine clinics within the Carilion system were willing to prescribe all fourteen of the proposed intervention modalities (i.e., from videos to in-person sessions). Providers were significantly less willing to prescribe weight loss medication when compared to the other 13 intervention types. The providers’ willingness to refer to a number of lifestyle interventions is encouraging for the integration of behavior change intervention in preconception care. Future work is needed to explore provider perceptions outside of rural Appalachia.

It is notable that, at large, providers share negative stereotypes of obesity [33]. Participants in the focus group remarked on these perceived judgments and the degree to which that made them distrust their physician and, in some cases, change providers. Therefore, it may be necessary in future efforts to build multi-level interventions, and to train providers on appropriate counseling related to weight loss prior to conception. Further, while providers’ physical activity recommendations supported the national guidelines, the dietary recommendations were less consistent, highlighting the need for additional provider training. Efforts should be made to ensure providers have a unified message for weight management with their patients.

### Limitations

The psychometric properties of many of the items used in this formative data collection have not undergone validity and reliability testing. Patient data was sufficiently powered to detect moderate to large effects for the likelihood-ratio chi-squared tests with two to eight degrees of freedom. Smaller effects may be present, but will require replication in larger samples to describe. This study was underpowered to detect differences in provider willingness to refer patients to different interventions, or perceptions of patients based on provider characteristics. Further, the anonymous nature of the provider survey made comparisons across clinical settings impossible. While provider characteristics may affect provider preferences and recommendations, limited cell counts precluded an analysis of provider outcomes according to provider characteristics in this study. Finally, the research team had difficulty recruiting and securing patient participants for the focus group. The minimum sample size recommendation to ensure saturation for qualitative data is five to six participants [34, 35]. Therefore, interpretations of the qualitative results need to be bolstered with future research to determine transferability. However, these results do provide a richer picture of patients’ preferences and perceptions than a report of the quantitative analyses alone. Encouragingly, despite the small sample size, the proportion of patients who were from low-income households providers support for the likelihood that preconception weight management efforts will reach patients with relevant health disparities.

## Conclusions

This is the first study, to our knowledge, to address both patients’ and providers’ perceptions of preconception weight management interventions. The results presented here suggest that a variety of intervention content, frequency, and duration would be well received by patients. In addition, providers would support a number of intervention types, favoring behavioral interventions over weight loss medications. Taken together, these findings suggest that an individualized weight management intervention can be implemented in an OBGYN setting as a form of preconception weight management, and would be well received by both patients and providers. Future research is needed to explore the feasibility and effect of preconception weight management interventions in sustained practice.

## Additional files

Additional file 1: Patient Survey. Survey distributed to patients for their perceptions of intervention content. (DOCX 187 kb)
Additional file 2: Provider survey. Survey distributed (electronically) to providers to capture their practices related to weight control and preconception interventions. (DOCX 150 kb)
Additional file 3: Semi-structured interview guide. Semi-structured interview guide for additional patient perceptions on preconception weight management. (DOCX 120 kb)

## Abbreviations

BMI: Body Mass Index
OBGYN: Obstetrics and Gynecology
