# Supplementary material for: Informing the development and uptake of a weight management intervention for preconception: a mixed-methods investigation of patient and provider perceptions
Source: BMC Obes. 2017 Feb 6;4:8. doi: 10.1186/s40608-017-0144-6 (PMC5295190; doi:10.1186/s40608-017-0144-6)
Supplement: Additional file 1: — Patient Survey. Survey distributed to patients for their perceptions of intervention content. (DOCX 187 kb) [file 40608_2017_144_MOESM1_ESM.docx]

Carilion Clinic OB/GYN

Patient Survey

As an OB/GYN patient between the ages of 21 and 35 at Carilion Clinic you are eligible to complete a brief survey.

Emily Hoeker-Evans, MD and Samantha Harden, PhD are investigating patient perceptions of weight status, gestational weight gain, and attributes of a program that may be attractive to women in Southwest Virginia.

All of the results will be reported as group data, whereby no individual will be identifiable in the results or any reports of the study findings. Any information you provide will remain anonymous. Please answer each question to the best of your knowledge. If you choose not to answer any question, just leave it blank and move on to the next question. **There are no right or wrong answers.**

**Risks:** Participation risks are minimal as information is anonymous (we will not collect any personal identifying information).

**Benefits**: Your participation in this survey will provide important data for the development and delivery of an evidence-based program for women in Southwest Virginia. This program may have a direct health and cost benefits for women and their infants.

Any questions or concerns about completing this survey can be directed to:

| Principal Investigator | **Institutional Review Board** |
| --- | --- |
| Emily Evans-Hoeker, MD  Reproductive Endocrinology & Infertility, Carilion Clinic  Department of OB/GYN  102 Highland Ave, Suite 304  Roanoke, VA 24013  540-985-8078 | 2001 Crystal Spring Avenue, Suite 202 Roanoke, VA 24014 540-853-0728 - See more at: http://www.carilionclinic.org/institutional-review-board/staff-roster#sthash.eylnQail.dpuf |

By completing this survey, you are providing consent to take part in this study. If you do not wish to complete the survey, you may return it to the front desk.

PART A: Tell us about you and your family.

1) **Pregnancy Status:**

🞏1 Planning to become pregnant within the year

🞏2 Currently pregnant

🞏3 Not planning to become pregnant in the next year

2) **Age** ________

3) **About how tall are you without shoes?**

______ feet _________ inches

4) **About how much do you weigh without shoes?**

____________ lbs

5) **My weight status is classified as:**

🞏1 Underweight

🞏2 Normal weight

🞏3 Overweight

🞏4 Obese

🞏5 Don’t know

**6) Please indicate which of the following best describes you:**

🞏1 White

🞏2 Black or African American

🞏3 Asian

🞏4 American Indian/Alaskan Native

🞏5 Native Hawaiian or Other Pacific Islander

🞏6 Not sure

🞏7 Other: _________

**7) Please indicate which of the following best describes you:**

🞏1 Hispanic or Latino

🞏2 Not Hispanic or Latino

🞏3 Not sure

**8) Please mark the highest grade of school that you have completed.**

🞏1 Grades 0-8

🞏2 Grades 9-11

🞏3 High school

🞏4 Some college

🞏5 College graduate

🞏6 Post college work

**9) What is your employment status?**

| 🞏1 Employed for wages | 🞏5 A homemaker |
| --- | --- |
| 🞏2 Self-Employed | 🞏6 A student |
| 🞏3 Out of work for more than 1 year | 🞏7 Retired |
| 🞏4 Out of work for less than 1 year | 🞏8 Disabled/unable to work |

**10) What was your total household income in 2013?**

🞏1 Less than $15,000

🞏2 $15,000 to $29,999

🞏3 $30,000 to $49,999

🞏4 $50,000 to $90,999

🞏5 $100,000 or more

**11) What is your marital status?**

🞏1 Single 🞏2 Married 🞏3 Widowed

🞏4 Separated 🞏5 Divorced

🞏6 Living common-law or living with partner

**12) About how tall is your significant other without shoes?**

______ feet _________ inches

**13) About how much does your significant other weigh without shoes?**

____________ lbs

**14) How many times have you been pregnant (including miscarriages and abortions)? __________**

**15) How many times have you had a pregnancy resulting in a live birth?**

**________**

**16) How much weight did you gain during your last pregnancy resulting in a live birth? _______lbs.**

**17) How long did it take you to get to your doctor’s visit today?**

🞏1 <10 minutes

🞏2 11-20 minutes

🞏3 21-40 minutes

🞏4 > 40 minutes

**18) How did you travel to your visit (e.g., walk, bus, car, someone drove you): _____________________________________________________________________________**

**Part B. Health**

**1) In general, compared to other persons your age, how would you rate your health?**

🞏1 Extremely healthy

🞏2 Somewhat healthy

🞏3 Not healthy

🞏4 Very unhealthy

🞏5 Don’t know

**2) How confident are you that you can engage in moderate physical activities (e.g., not exhausting, light perspiration) for 30 minutes for 5 or more days per week?**

🞏1 Not at all 🞏2 Somewhat 🞏3 Moderately 🞏4Very 🞏5 Completely

**3) Physical activity over the past week.**

Considering the past 7-day period (last week), how many times did you do the following kinds of exercise for more than 15 minutes during your free time (write on each line the appropriate number)? Only count exercise that was done during free time (i.e., not occupation or housework). Note that the main difference between the three categories is the intensity of the exercise.

Please write the average frequency on the first line and the average duration on the second line.

**Times Per Week Average Duration**

**(Minutes Per Session)**

a. STRENUOUS EXERCISE __________ __________

(HEART BEATS RAPIDLY, SWEATING)

(e.g., running, jogging, hockey, soccer, squash, cross

country skiing, vigorous swimming, vigorous long

distance bicycling, vigorous aerobic dance classes,

heavy weight training)

b. MODERATE EXERCISE __________ __________

(NOT EXHAUSTING, LIGHT PERSPIRATION)

(e.g., fast walking, baseball, tennis, easy bicycling,

volleyball, badminton, easy swimming, alpine skiing,

popular and folk dancing)

c. MILD EXERCISE __________ __________

(MINIMAL EFFORT, NO PERSPIRATION)

(e.g., easy walking, yoga, bowling)

**4) What are the physical activity recommendations for most adults?**

🞏1 30 minutes of moderate intensity physical activity 5 days a week

🞏2 20 minutes of moderate intensity physical activity 3 times per week

🞏3 60 minutes of moderate intensity physical activity most days of the week

🞏4 Unsure

**5) The amount of physical activity I engage in is:**

🞏1 Less than the recommended amount of physical activity

🞏2 Meeting physical activity recommendations

🞏3 More than the physical activity recommendations

🞏4 I do not engage in physical activity

🞏5 Unsure

**
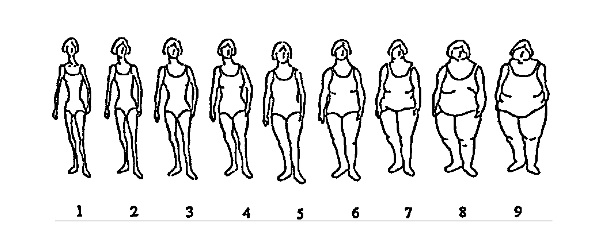
6) Please circle the image that best reflects your body size.**

**Part C. Questions related to body weight prior to pregnancy.**

**1) If I were planning to become pregnant (but was not yet pregnant), I should:**

🞏1 Lose weight

🞏2 Stay the same weight

🞏3 Gain weight

🞏4 Unsure

**2) If I were planning to become pregnant (but was not yet pregnant), I should:**

🞏1 Increase my physical activity level

🞏2 Continue the same physical activity level

🞏3 Decrease my physical activity level

🞏4 Unsure

**3) If I were planning to become pregnant (but was not yet pregnant), I should:**

🞏1 Increase how much I eat

🞏2 Continue eating the same amount

🞏3 Decrease how much I eat

🞏4 Unsure

**4) Being overweight or obese before pregnancy increases my risks of:**

| Experiencing difficulties conceiving | 🞏1 **True** | 🞏2**False** | 🞏3 **Unsure** |
| --- | --- | --- | --- |
| Pregnancy related complications | 🞏1 **True** | 🞏2 **False** | 🞏3 **Unsure** |
| Having a difficult child birth | 🞏1 **True** | 🞏2 **False** | 🞏3 **Unsure** |
| My child being overweight or obese | 🞏1 **True** | 🞏2 **False** | 🞏3 **Unsure** |

**Part D. Weight During Pregnancy**

1. **How much weight should you gain during pregnancy?**

|  | ≤ 5 lbs | 6-10 lbs | 11-15 lbs | 16-20 lbs | 21-25 lbs | 26-30 lbs | 31-35 lbs | 36-40 lbs | Unsure |
| --- | --- | --- | --- | --- | --- | --- | --- | --- | --- |
| Months 1-3 |  |  |  |  |  |  |  |  |  |
| Months 4-6 |  |  |  |  |  |  |  |  |  |
| Months 7-9 |  |  |  |  |  |  |  |  |  |
| Total weight gain during pregnancy |  |  |  |  |  |  |  |  |  |

**2) The amount of weight I gain during pregnancy depends on my pre-pregnancy weight status.**

🞏1 True

🞏2 False

🞏3 Unsure

**3) During pregnancy I should:**

🞏1 Increase my physical activity level

🞏2 Continue the same physical activity level

🞏3 Decrease my physical activity level

🞏4 Unsure

4) **During pregnancy I should:**

🞏1 Increase how much I eat

🞏2 Continue eating the same amount.

🞏3 Decrease how much I eat

🞏4 Unsure

**5) During pregnancy, you should engage in physical activity, unless told otherwise by your physician**

🞏1 True

🞏2 False

🞏3 Unsure

**6) Gaining more weight than recommended during pregnancy increases my risks of:**

| Pregnancy related complications | 🞏1 True | 🞏2 False | 🞏3Unsure |
| --- | --- | --- | --- |
| Having a difficult child birth | 🞏1 True | 🞏2 False | 🞏3Unsure |
| My child being overweight or obese | 🞏1 True | 🞏2 False | 🞏3Unsure |
| Retaining excess weight after delivery | 🞏1 True | 🞏2 False | 🞏3Unsure |

Part E. Intervention Components

We are working to develop an appropriate pre-pregnancy weight control program for patients in Carilion Clinic OB/GYN.

**1) I would regularly attend a health promotion class that met:**

*(Please select all that apply)*

🞏1 3 times per week 🞏2 Weekly 🞏3 Monthly 🞏4 Would not attend

**2) I would regularly attend a health promotion class that met for:**

*(Please select all that apply)*

🞏1 30 minutes 🞏2 60 minutes 🞏3 90 minutes 🞏4 Would not attend

**3) I would regularly attend a health promotion class that provided information:**

*(Please select all that apply)*

🞏1 In-person 🞏2 Online 🞏3 Via email 🞏4 Via DVD/Video

🞏5 Via text message 🞏6 Would not attend

**4) I would regularly attend a health promotion class that encouraged me to exercise:**

*(Please select all that apply)*

🞏1 At home 🞏2 In a gym 🞏3 With an online coach

🞏4 In a group fitness class 🞏5 Would not attend

**5) I would regularly attend a health promotion class that included exercising during the session.**

🞏1 Yes 🞏2 No 🞏3 Unsure

**6) I would regularly attend a health promotion class that met:**

*(Please select all that apply)*

🞏1 At a hospital 🞏2 In a gym 🞏3 In an office 🞏4 Community room

🞏5 Other__________________________ 🞏6 Would not attend

**7) I would need an incentive (e.g., gift card, door prize) to attend a health promotion class.**

*(Please select all that apply)*

🞏1 Strongly Agree

🞏2 Agree

🞏3 Neither agree nor disagree

🞏4 Disagree

🞏5 Strongly disagree

**8) The following program characteristics would be appealing to me in a health promotion program. (Please check all that apply).**

| 🞏 Tracking my progress | 🞏 Healthy recipes | 🞏 Opportunities to interact with others in the group |
| --- | --- | --- |
| 🞏 Developing goals | 🞏 Cooking demonstrations | 🞏 One-on-one counselling with a health professional |
| 🞏 A credible exercise instructor | 🞏 Tips for cheap, healthy eating | 🞏 Opportunities to discuss barriers to success with a health professional |
| 🞏 A peer exercise instructor (similar age, same sex) | 🞏 Grocery store demonstration (e.g., tour for healthy shopping) | 🞏 Opportunities to discuss barriers to success with other women trying to lose weight |
| 🞏 Feedback on my goals | 🞏 Assistance with portion control | 🞏 Exercising alone |
| 🞏 Exercise diary  🞏 In-person sessions | 🞏 Food diary  🞏 Online sessions | 🞏 Exercising in a group  🞏 Medication for weight loss |

If you are planning to become pregnant or are currently pregnant:

Are you interested in participating in a focus group (group interview) with the purpose of finding out more information about this topic. The research team is going to ask questions related to your perceptions of excess weight prior to and during pregnancy; however, the majority of the interview will focus on the development and design of a program for women to achieve a healthy weight status prior to pregnancy. You will be provided lunch as well as monetary compensation for participating in this focus group.

If you are interested, please fill out the form below, remove this page, and return separately to the front desk.

**Name**: ______________________________________________________________

**Age**: ____________

**Phone Number:**

**Email Address:**

**Preferred method of contact**:

**Dietary Restrictions:** ______________________________________________________________

______________________________________________________________

______________________________________________________________

**Raffle Drawing**

If you would like to be entered to win one of 20 $50 prizes, please complete the following information and return to the box at the front desk.

Name:

Address:

Phone: ­

If you are randomly selected, your prize will arrive by certified mail. It will also include a receipt for you to sign and return in a pre-addressed envelope.
