# Supplementary material for: Informing the development and uptake of a weight management intervention for preconception: a mixed-methods investigation of patient and provider perceptions
Source: BMC Obes. 2017 Feb 6;4:8. doi: 10.1186/s40608-017-0144-6 (PMC5295190; doi:10.1186/s40608-017-0144-6)
Supplement: Additional file 2: — Provider survey. Survey distributed (electronically) to providers to capture their practices related to weight control and preconception interventions. (DOCX 150 kb) [file 40608_2017_144_MOESM2_ESM.docx]

Carilion Clinic OB/GYN

Provider Survey

Emily Evans-Hoeker, MD and Samantha Harden, PhD are investigating OB/GYN provider current practices in regards to weight status and gestational weight gain, and attributes of a program that may be feasible to implement in your daily practice.

All of the results will be reported as group data, whereby no individual will be identifiable in the results or any reports of the study findings. Any information you provide will remain anonymous. Please answer each question to the best of your knowledge. If you choose not to answer any question, just leave it blank and move on to the next question. **There are no right or wrong answers.**

**Risks:** Participation risks are minimal as information is anonymous on the survey.

**Benefits**: Your participation in this survey will provide important data for the development and delivery of an evidence-based program for women in Southwest Virginia. This program may have direct health and cost benefits for women and their infants. **If you complete at least 90% of the survey you will have the opportunity to enter into a drawing to win one of five $100 gift cards.**

Any questions or concerns about completing this survey can be directed to:

| Principal Investigator | **Institutional Review Board** |
| --- | --- |
| Emily Evans-Hoeker, MD  Reproductive Endocrinology & Infertility, Carilion Clinic  Department of OB/GYN  102 Highland Ave, Suite 304  Roanoke, VA 24013  [540-985-8078](tel:540-985-8078) | 2001 Crystal Spring Avenue, Suite 202 Roanoke, VA 24014 540-853-0728 - See more at: http://www.carilionclinic.org/institutional-review-board/staff-roster#sthash.eylnQail.dpuf |

By completing this survey, you are providing consent to take part in this study. If you do not wish to complete the survey, you may exit the survey by closing your browser. This survey is voluntary and will not affect your job or status in any way.

**PART A: Tell us about you.**

1) **What type of provider are you?**

🞏1 Attending physician

🞏2 Resident physician

🞏3 Nurse practitioner

🞏4 Other____________

2) **How many years have you worked for Carilion? _____________________________**

3) **Where were you before you began working for Carilion? (check all that apply)**

🞏1 In training

🞏2 In private practice

🞏3 In academic practice

🞏4 In an academic-private hybrid practice

🞏5 In a different career

🞏6 Other: ______________________________________________________________

4) **What is your sex?**

🞏1 Male

🞏2 Female

5) **What is your age?** ________

6) **About how tall are you without shoes?**

______ feet _________ inches

7) **About how much do you weigh without shoes?**

____________ lbs

8) **Please indicate which of the following best describes you:**

🞏1 White

🞏2 Black or African American

🞏3 Asian

🞏4 American Indian/Alaskan Native

🞏5 Native Hawaiian or Other Pacific Islander

🞏6 Not sure

🞏7 Other: _________

9) **Please indicate which of the following best describes you:**

🞏1 Hispanic or Latino

🞏2 Not Hispanic or Latino

🞏3 Not sure

**PART B: This section is meant to inform us of current practices in your office in regards to your gynecology patients. Please answer the following questions in regards to your NON-PREGNANT, 21-35 YEAR OLD PATIENTS.**

| **How often do you…** | Always | Often | Sometimes | Rarely | Never | N/A |
| --- | --- | --- | --- | --- | --- | --- |
| review your patient’s BMI during new patient visits? |  |  |  |  |  |  |
| review your patient’s BMI during follow-up visits? |  |  |  |  |  |  |
| inform your patient of her BMI/weight status? |  |  |  |  |  |  |
| ask about your patient’s plans to conceive within the next 12 months? |  |  |  |  |  |  |
| ask about your patients physical activity? |  |  |  |  |  |  |
| make specific physical activity recommendations? |  |  |  |  |  |  |
| ask about your patients diet? |  |  |  |  |  |  |
| make specific diet recommendations? |  |  |  |  |  |  |
| prescribe weight loss medications? |  |  |  |  |  |  |
| counsel your overweight and/or obese patients planning conception in the next 12 months that they should lose weight prior to conception based on their BMI? |  |  |  |  |  |  |
| provide specific physical activity recommendations to patients planning conception within the next 12 months? |  |  |  |  |  |  |
| provide specific diet recommendations to patients planning conception within the next 12 months? |  |  |  |  |  |  |
| counsel your overweight or obese patients that they should not conceive due to their BMI? |  |  |  |  |  |  |
| decline to provide fertility assistance (including ovulation induction agents) to your overweight and/or obese patients because of their BMI? |  |  |  |  |  |  |
|  |  |  |  |  |  |  |
| refer your overweight/obese patients to another provider to assist with weight loss? |  |  |  |  |  |  |
| prescribe FitRx? |  |  |  |  |  |  |
| refer your patients with a BMI of ≥ 35 to bariatric surgery? |  |  |  |  |  |  |
| counsel your patients who are contemplating conception within the next 12 months that being overweight or obese at the time of conception increases the risk of the following: |  |  |  |  |  |  |
|  |  |  |  |  |  |  |
| infertility |  |  |  |  |  |  |
| pregnancy related complications |  |  |  |  |  |  |
| delivery complications |  |  |  |  |  |  |
| childhood obesity |  |  |  |  |  |  |

1) **How would you rate the health of** your NON-PREGNANT, 21-35 YEAR OLD PATIENTS?

🞏1 Extremely healthy

🞏2 Somewhat healthy

🞏3 Not healthy

🞏4 Very unhealthy

🞏5 Don’t know

2) **How confident are you that they are physically able to engage in moderate physical activities for 30 min, 5 or more days per week**?

🞏1 Not at all 🞏2 Somewhat 🞏3 Moderately 🞏4Very 🞏5 Completely

3) **In your NON-PREGNANT, 21-35 YEAR OLD PATIENTS what are your typical recommendations for:**

a) Physical activity _______________________________________________________

b) Diet _________________________________________________________________

4) **Do your physical activity recommendations change if your patient is planning conception within the next 12 months?**

🞏1 No

🞏2 Yes, in general I recommend decreased physical activity

🞏3 Yes, in general I recommend increased physical activity

🞏4 I don’t make recommendations for physical activity

5) **Which weight loss medications do you prescribe? (check all that apply)**

🞏1 None

🞏2 Orlistat (Alli, Xenical)

🞏3 Metformin

🞏4 Bupropion (Wellbutrin, Zyban)

🞏5 Phentermine (Adipex)

🞏6 Lorcaserin (Belviq)

🞏7 Phentermine-Topiramate (Qsymia)

🞏8 Bupropion-Naltrexone (Contrave)

🞏9 Benzphetamine (Didrex)

🞏10 Diethylpropion (Tenuate)

🞏11 Phendimetrazine (Adipost)

🞏12 Other ________________________

6) **At what BMI do you recommend weight loss prior to conception? (check all that apply)**

🞏1 Overweight (BMI 25-29.9)

🞏2 Obesity I (BMI 30-34.9)

🞏3 Obesity II (BMI 35-39.9)

🞏4 Obesity III (BMI ≥ 40)

🞏5 I do not typically recommend weight loss prior to conception

🞏6 Other ________________________

7) **Who do you typically refer your patients to for assistance in weight loss? (check all that apply)**

🞏1 Nutritionist or registered dietician

🞏2 Exercise physiologist or personal trainer

🞏3 Psychologist or psychiatrist

🞏4 Family practice or internal medicine

🞏5 Bariatric surgeon

🞏6 Endocrinologist

🞏7 Commercial weight loss program (i.e. weight watchers, Jenny Craig, etc)

🞏8 Community support group

🞏9 I do not typically refer my patients to other providers to assist in weight loss

🞏10 I do know who I can refer my patients to for assistance in weight loss

🞏11 Other __________________________________________________

**PART C: This section is meant to inform us of current practices in your office in regards to your obstetric patients. Please answer the following questions in regards to your LOW-RISK, PREGNANT PATIENTS.**

| **How often do you…** | Always | Often | Sometimes | Rarely | Never | N/A |
| --- | --- | --- | --- | --- | --- | --- |
| review your patients gestational weight gain during new patient visits? |  |  |  |  |  |  |
| review your patients gestational weight gain during follow up visits? |  |  |  |  |  |  |
| inform patients of their gestational weight gain? |  |  |  |  |  |  |
| inform pregnant patients of the gestational weight gain recommendations? |  |  |  |  |  |  |
| inform patients that their weight gain is or is not within recommendations? |  |  |  |  |  |  |
| ask about the patients physical activity during pregnancy? |  |  |  |  |  |  |
| make specific physical activity recommendations to pregnant patients? |  |  |  |  |  |  |
| ask about the patients diet during pregnancy? |  |  |  |  |  |  |
| make specific diet recommendations to pregnant patients (for healthy weight purposes, not including infectious and mercury diet precautions)? |  |  |  |  |  |  |
| refer pregnant patients to another provider to assist with preventing excess gestational weight gain? |  |  |  |  |  |  |
| prescribe FitRx to pregnant patients? |  |  |  |  |  |  |
| counsel patients who are pregnant that excess gestational weight gain increases the risk of the following: |  |  |  |  |  |  |
| pregnancy related complications |  |  |  |  |  |  |
| delivery complications |  |  |  |  |  |  |
| childhood obesity |  |  |  |  |  |  |
| retaining excess weight after delivery |  |  |  |  |  |  |

1) **How would you rate the health of your LOW-RISK, PREGNANT PATIENTS?**

🞏1 Extremely healthy

🞏2 Somewhat healthy

🞏3 Not healthy

🞏4 Very unhealthy

🞏5 Don’t know

2) **How confident are you that they are physically able to engage in moderate physical activities for 30 min, 5 or more days per week**?

🞏1 Not at all 🞏2 Somewhat 🞏3 Moderately 🞏4Very 🞏5 Completely

3) **In your** **LOW-RISK, PREGNANT PATIENTS what are your typical recommendations for physical activity?**

🞏1 In general I recommend decreased physical activity

🞏2 In general I recommend increased physical activity

🞏3 In general I recommend they continue with their current physical activity

🞏4 I don’t make recommendations for physical activity

4) **Please complete the below table regarding your gestational weight gain recommendations in your** **LOW-RISK, PREGNANT PATIENTS based upon pre-pregnancy weight status and by trimester:**

|  | ≤ 5 lbs | 6-10 lbs | 11-15 lbs | 16-20 lbs | 21-25 lbs | 26-30 lbs | 31-35 lbs | 36-40 lbs | I don’t make specific recommendations |
| --- | --- | --- | --- | --- | --- | --- | --- | --- | --- |
| **For normal weight pregnant patients** |  |  |  |  |  |  |  |  |  |
| 1st trimester |  |  |  |  |  |  |  |  |  |
| 2nd trimester |  |  |  |  |  |  |  |  |  |
| 3rd trimester |  |  |  |  |  |  |  |  |  |
| total weight gain |  |  |  |  |  |  |  |  |  |
| **For overweight pregnant patients** |  |  |  |  |  |  |  |  |  |
| 1st trimester |  |  |  |  |  |  |  |  |  |
| 2nd trimester |  |  |  |  |  |  |  |  |  |
| 3rd trimester |  |  |  |  |  |  |  |  |  |
| total weight gain |  |  |  |  |  |  |  |  |  |
| **For obese pregnant patients** |  |  |  |  |  |  |  |  |  |
| 1st trimester |  |  |  |  |  |  |  |  |  |
| 2nd trimester |  |  |  |  |  |  |  |  |  |
| 3rd trimester |  |  |  |  |  |  |  |  |  |
| total weight gain |  |  |  |  |  |  |  |  |  |

5) **Who do you refer your patients to for assistance in preventing excess gestational weight gain? (check all that apply)**

🞏1 Nutritionist or registered dietician

🞏2 Exercise physiologist or personal trainer

🞏3 Psychologist or psychiatrist

🞏4 Family practice or internal medicine

🞏5 Endocrinologist

🞏6 Community support group

🞏7 I do not typically refer my patients to other providers to assist in preventing excess gestational weight gain

🞏8 I do know who I can refer my patients to for assistance in preventing excess gestational weight gain

🞏9 Other __________________________________________________

**PART D: This portion of the survey is to assess feasibility of potential weight loss interventions in your current ob/gyn practice. Please indicate how strongly you agree with the following statements in regards to both your NON-PREGNANT, 21-35 YEAR OLD PATIENTS as well as your LOW-RISK, PREGNANT PATIENTS.**

|  | NON-PREGNANT, 21-35 YEAR OLD PATIENTS | | | | |  | LOW-RISK, PREGNANT PATIENTS | | | | |
| --- | --- | --- | --- | --- | --- | --- | --- | --- | --- | --- | --- |
|  | Strongly Agree | Agree | Neutral | Disagree | Strongly Disagree |  | Strongly Agree | Agree | Neutral | Disagree | Strongly Disagree |
| I have time discuss weight during my patient visits |  |  |  |  |  |  |  |  |  |  |  |
| I feel comfortable discussing my patients’ weight during their visits |  |  |  |  |  |  |  |  |  |  |  |
| I would be willing to discuss weight during my visits |  |  |  |  |  |  |  |  |  |  |  |
| I have time to discuss physical activity recommendations during my visits |  |  |  |  |  |  |  |  |  |  |  |
| I feel comfortable discussing physical activity recommendations during my visits |  |  |  |  |  |  |  |  |  |  |  |
| I would be willing to discuss physical activity recommendations during my visits |  |  |  |  |  |  |  |  |  |  |  |
| I am willing to prescribe weight loss medications to my patients |  |  |  |  |  |  |  |  |  |  |  |
| I would be willing to prescribe weight loss medications if I had more education/training regarding their use |  |  |  |  |  |  |  |  |  |  |  |
| I am not willing to prescribe weight loss medications to my patients regardless of education that may be available regarding the use of weight loss medications |  |  |  |  |  |  |  |  |  |  |  |
| I would prefer someone else discuss weight with my patients |  |  |  |  |  |  |  |  |  |  |  |
| My patients do not need a weight loss discussion |  |  |  |  |  |  |  |  |  |  |  |
| I would be **willing to encourage** my patients to participate in the following: |  |  |  |  |  |  |  |  |  |  |  |
| watch an informational video in the office after the visit is completed |  |  |  |  |  |  |  |  |  |  |  |
| join a community group program |  |  |  |  |  |  |  |  |  |  |  |
| receive electronic (online) education about weight |  |  |  |  |  |  |  |  |  |  |  |
| receive weight related education via their mobile phone (via app or text) |  |  |  |  |  |  |  |  |  |  |  |
| receive an individualized diet/activity plan |  |  |  |  |  |  |  |  |  |  |  |
| receive a behavioral change plan |  |  |  |  |  |  |  |  |  |  |  |
| see someone else within Carilion to manage weight |  |  |  |  |  |  |  |  |  |  |  |
| join a commercial weight loss program (e.g., weight watchers) |  |  |  |  |  |  |  |  |  |  |  |
| take weight loss medications (can be prescribed by another provider) |  |  |  |  |  |  |  |  |  |  |  |
| undergo weight loss surgery |  |  |  |  |  |  |  |  |  |  |  |
| The following interventions **would be helpful** in achieving weight loss in my patient population: |  |  |  |  |  |  |  |  |  |  |  |
| watch an informational video in the office after the visit is completed |  |  |  |  |  |  |  |  |  |  |  |
| join a community group program |  |  |  |  |  |  |  |  |  |  |  |
| receive electronic (online) education about weight |  |  |  |  |  |  |  |  |  |  |  |
| receive weight related education via their mobile phone (via app or text) |  |  |  |  |  |  |  |  |  |  |  |
| receive an individualized diet/activity plan |  |  |  |  |  |  |  |  |  |  |  |
| receive a behavioral change plan |  |  |  |  |  |  |  |  |  |  |  |
| see someone else within Carilion to manage weight |  |  |  |  |  |  |  |  |  |  |  |
| join a commercial weight loss program (i.e. weight watchers) |  |  |  |  |  |  |  |  |  |  |  |
| take weight loss medications (can be prescribed by another provider) |  |  |  |  |  |  |  |  |  |  |  |
| undergo weight loss surgery |  |  |  |  |  |  |  |  |  |  |  |
| watch an informational video in the office after the visit is completed |  |  |  |  |  |  |  |  |  |  |  |

Thank you for completing our survey!

If you are interested in entering to win one of five $100 gift cards please enter your email below:

Email: ______________________________________________________________________
