# Supplementary material for: Informing the development and uptake of a weight management intervention for preconception: a mixed-methods investigation of patient and provider perceptions
Source: BMC Obes. 2017 Feb 6;4:8. doi: 10.1186/s40608-017-0144-6 (PMC5295190; doi:10.1186/s40608-017-0144-6)
Supplement: Additional file 3: — Semi-structured interview guide. Semi-structured interview guide for additional patient perceptions on preconception weight management. (DOCX 120 kb) [file 40608_2017_144_MOESM3_ESM.docx]

**Preconception Weight Loss Intervention**

**Focus Group Script**

Thank you for joining today to participate in the Carilion Clinic OB/GYN focus group. A focus group is an interview with multiple persons at once in order to gain a rich understanding of a particular topic. Today we want to discuss your perception of the effect of excess weight prior to conception and excess weight gain during pregnancy as well as opinions on the best ways to assist women with achieving healthy weight status prior to conception. There will be approximately 20 subjects from Southwest Virginia Carilion Clinic taking part. The length of time you can expect to be in this research is 90 minutes.

I am _______, and I am part of the research team. We will start the day by talking about your role as a participant in this study, obtaining informed consent, completing a brief questionnaire and grabbing lunch. Then, I will start the focus group. My role as a facilitator is to present the topic areas, probe for any follow-up details we may need related to a specific response, and to keep track of time.

______ is here as my assistant to take notes and help me keep track of time.

First I will read the consent form, allow time for any related questions, and collect signed consent for those of you who wish to continue with this focus group interview.

*(Read consent form, collect signed consent forms. Participants grab lunch and complete questionnaire. Resume [~15 minutes]).*

There are no right or wrong answers, so please share your experience and thoughts as we continue.

I will begin recording now.

Please state the number assigned to you in your packet.

(Allow participants to read their assigned participant number to both test for volume and ‘record’ voice recognition)

Thank you for joining.

**Our first few questions are related to body weight prior to pregnancy.**

*Attitude (not read)*

Please tell me about the positive things that may happen if you were to lose weight prior to getting pregnant.

Please tell me about the negative things that may happen if you were to lose weight prior to getting pregnant.

*Subjective Norms (not read)*

Health professionals recommend that women be at a healthy weight status prior to conception. This would be a body mass index (or BMI) of 21-24.9. You determine your BMI by your height and weight. *Everyone has a BMI chart in her folder, please find the weight you should be based on your height.* Tell me how you feel about this recommendation.

What would it take for someone to convince you that it is important to lose weight prior to pregnancy?

Probes: who, how, why

*Behavioral Intention (not read)*

Please describe your intentions to lose weight prior to pregnancy?

*Implementation Intention (not read)*

If you plan to lose weight prior to conception, how would you do so?

*Probes: when, where, how*

*Perceived Behavioral Control (not read)*

What would make it easy to lose weight prior to pregnancy?

What would make it hard to lose weight prior to pregnancy?

What would you need to help you lose weight prior to pregnancy?

**The next few questions are related to healthy eating and physical activity prior to pregnancy.**

*Attitude (not read)*

Please tell me about the positive things that may happen if you exercise.

Please tell me about the negative things that may happen if you exercise.

Please tell me about the positive things that may happen if you eat healthfully.

Please tell me about the negative things that may happen if you healthfully.

How do you know if you are eating healthfully?

*Subjective Norms (not read)*

Health professionals recommend that women are active for 30 minutes most days of the week. Tell me how you feel about this recommendation.

What would it take for someone to convince you that it is important to be active for 30 minutes most days of the week?

Probe: who, how, why

Health professionals recommend that women eat 2.5 cups of vegetables a day. *(show 2.5 cups of vegetables).* Tell me how you feel about this recommendation.

What would it take for someone to convince you that it is important to eat 2.5 cups of vegetables a day?

Probe: who, how, why

*Behavioral Intention (not read)*

Please describe your intentions to be active for 30 minutes most days of the week prior to pregnancy?

Please describe your intentions to eat 2.5 cups of vegetables prior to pregnancy?

*Implementation Intention (not read)*

If you plan to be active prior to conception, how would you do so?

*Probes: when, where, how*

If you plan to eat 2.5 cups of vegetables prior to conception, how would you do so?

*Probes: when, where, how*

If you already meet these recommendations, how will you maintain these behaviors?

*Perceived Behavioral Control (not read)*

What would make it easy to meet physical activity recommendations?

What would make it hard to meet physical activity recommendations?

What would you need to meet physical activity recommendations?

What would make it easy to meet vegetable intake recommendations?

What would make it hard to meet vegetable intake recommendations?

What would you need to meet vegetable intake recommendations?

**The next few questions are related to developing a program for weight loss prior to pregnancy.**

We are working to develop an appropriate pre-pregnancy weight control program for patients in Carilion Clinic OB/GYN.

How do you feel about an in-person health promotion class?

Probe: what do you want the class to focus on?, have you ever attended a similar class? If so, what were your experience?

How often should the class meet?

How long should the class be?

Where would it be convenient for the classes to meet?

Town:

Building type:

*Probe: What is your commute to Roanoke?*

What would you do in this class?

How do you feel about health promotional information delivered via:

DVD

Text

Online

Email

In-person

What program characteristics would you prefer to see in a health promotion class?

*Probes: recipes, tracking, in a group, one-on-one, cooking demonstration, feedback on goals, diaries,*

**Wrap-Up (Remaining Time)**

Is there anything else you would like to share with the research team at this time?

**I am going to stop recording now.**

Thank you for your participation in this focus group. Members of the research team will transcribe these sessions verbatim (or, word for word). We will then interpret the findings in order to develop a program for Carilion Clinic patients. If at any time during this process you wish to retract all or part of your statements, you may do so. You have our contact information on your consent form documents.
